# Dealloyed Porous NiFe_2_O_4_/NiO with Dual-Network Structure as High-Performance Anodes for Lithium-Ion Batteries

**DOI:** 10.3390/ijms24044152

**Published:** 2023-02-19

**Authors:** Chao Jin, Zigang Wang, Chang Luo, Chunling Qin, Yongyan Li, Zhifeng Wang

**Affiliations:** 1 School of Materials Science and Engineering, Hebei University of Technology, Tianjin 300401, China; 2 Key Laboratory for New Type of Functional Materials in Hebei Province, Hebei University of Technology, Tianjin 300401, China

**Keywords:** NiFe_2_O_4_, dealloying, porous, lithium-ion batteries

## Abstract

As high-capacity anode materials, spinel NiFe_2_O_4_ aroused extensive attention due to its natural abundance and safe working voltage. For widespread commercialization, some drawbacks, such as rapid capacity fading and poor reversibility due to large volume variation and inferior conductivity, urgently require amelioration. In this work, NiFe_2_O_4_/NiO composites with a dual-network structure were fabricated by a simple dealloying method. Benefiting from the dual-network structure and composed of nanosheet networks and ligament-pore networks, this material provides sufficient space for volume expansion and is able to boost the rapid transfer of electrons and Li ions. As a result, the material exhibits excellent electrochemical performance, retaining 756.9 mAh g^−1^ at 200 mA g^−1^ after cycling for 100 cycles and retaining 641.1 mAh g^−1^ after 1000 cycles at 500 mA g^−1^. This work provides a facile way to prepare a novel dual-network structured spinel oxide material, which can promote the development of oxide anodes and also dealloying techniques in broad fields.

## 1. Introduction

Green and sustainable lithium-ion batteries (LIBs) have been widely applied in various energy storage systems, particularly in luggable electronic devices [1,2]. Driven by the requirement for environmental protection and large-scale energy storage (such as smart electric grids and electric vehicles), the performance of LIBs should be enhanced urgently [2,3,4]. High specific capacity, fast rate property, and the long-term lifespan of anode materials are key factors for obtaining commercial LIBs with high energy density [5]. Traditional graphite anodes possess a low theoretical capacity (372 mAh g^−1^), which cannot satisfy growing market requirements. Owing to higher theoretical capacity (700–1100 mAh g^−1^) than graphite anodes [6], transition metal oxide (TMOs) have been proven as potential materials for LIBs. Particularly, (inverse) spinel NiFe_2_O_4_, a kind of binary transition-metal oxides, has aroused extensive attention from researchers for its high theoretical capacity (915 mAh g^−1^), natural abundance, and safe working voltage [7,8]. Nonetheless, NiFe_2_O_4_ materials undergo relatively large volume variations during lithiation/delithiation cycling, which are accompanied by the influence of diffusion-induced stresses [9,10]. These stresses can cause mechanical damage to the electrode (deformation and fracture), which leads to disconnects between the NiFe_2_O_4_ and current collectors and ultimately causes capacity fade [9,11,12].

There are many efforts to alleviate the drawbacks of NiFe_2_O_4_ materials in LIBs. Nano-structuration toward materials is an effective approach. NiFe_2_O_4_ has been fabricated into nanoparticles, nanowires, nanotubes, nanosheets, etc. [13,14]. These nanoscale materials can shorten the diffusion distance of ions/electrons for fast charging/discharge and provide more active reaction sites for enhanced capacity [6,15]. For example, Wang et al. [13] synthesized one-dimensional NiFe_2_O_4_ nanofibers, yolk/shell nanofibers, and nanotubes by an electrospinning and waterpower stirring method. The NiFe_2_O_4_ nanotubes exhibited a stabilized capacity of 1349 mAh g^−1^ at 100 mA g^−1^ after 220 cycles. However, the preparation process needed sophisticated techniques accompanied by a high voltage supply and heat treatment. Furthermore, large volume changes cannot be effectively ameliorated by these nano-sized materials alone during prolonged high-rate cycling [16,17]. The construction of hierarchical porous nanostructures is a common method to address this problem. For instance, Wei et al. [18] fabricated hierarchical porous NiFe_2_O_4_ nanosheets by using waste bagasse as a template. The reversible capacity of this anode remained at 1229.1 and 989.6 mAh g^−1^ at 200 and 500 mA g^−1^ after 300 cycles, respectively. Meanwhile, the porous NiFe_2_O_4_ materials presented excellent rate performance and structural stability. Hierarchical porous nanostructures not only provide sufficient buffer space in the electrode to accommodate drastic volume changes but also facilitate electrolyte diffusion and electron transport [19,20]. However, the general process of constructing hierarchical porosity requires complex crafts, for instance, the template method, which is usually further treated by acidic erosion and/or heat treatment [18]. These complex technologies, including environmentally unfriendly and energy-intensive processes, limit the further application of NiFe_2_O_4_ as LIB anodes.

The dealloying method is a common process that involves building up a three-dimensional nanoporous structure [20,21,22]. During the dealloying process, the reaction active elements or compositions are selectively removed through chemical or electrochemical processes, and residual components spontaneously migrate and diffuse to form unique nanoporous structures [22]. The dealloying method has appealed much attention in the research community because of its low spending, scalable fabrication, and controllable product morphology [22,23]. The aim of this study was to explore the unique porous structure created by dealloying technology and to develop high-performance NiFe_2_O_4_-based anodes for LIB application. Herein, we designed a NiFe_2_O_4_/NiO composite with a dual-network structure by the facile processing and environmental friendliness dealloying method as well as carefully designing precursor components. The dual networks include a nanosheet network and a ligament-pore (bimodal porous) network with abundant oxygen vacancies and active sites, exhibiting good Li storage properties as LIB anode materials. This work provides a novel strategy to fabricate spinel TMOs and can promote the development of dealloying techniques in broad fields.

## 2. Results and Discussion

**Figure 1:**
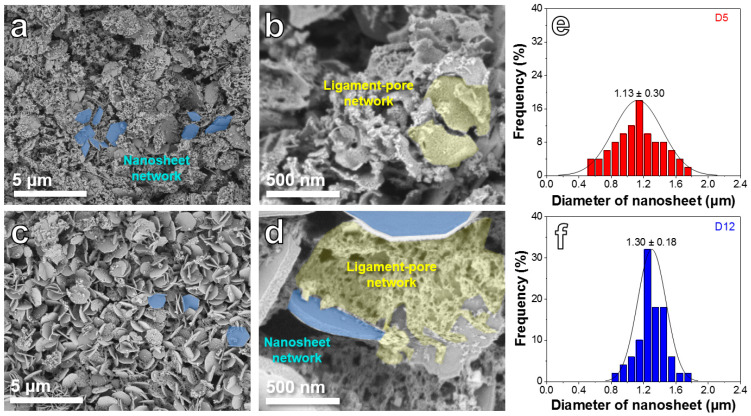
SEM images of D5 (**a**,**b**) and D12 (**c**,**d**). Nanosheet diameter distribution of D5 (**e**) and D12 (**f**).

Figure 1a,b reveals the SEM images of the D5 sample, which shows a dual-network structure, including a nanosheet network and a ligament-pore network. A large number of pores can be seen on ligamental skeletons (Figure 1b). As presented in Figure 1c,d, the D12 sample also possesses the dual-network structure, while the volume ratio of the ligament-pore network to the nanosheet network is obviously lower than the D5 sample. Furthermore, the nanosheet diameter distribution features of the two samples are uncovered in Figure 1e,f. The D5 sample exhibits a smaller peak-value nanosheet diameter (1.13 ± 0.30 μm) than the D12 sample (1.30 ± 0.18 μm).

**Figure 2:**
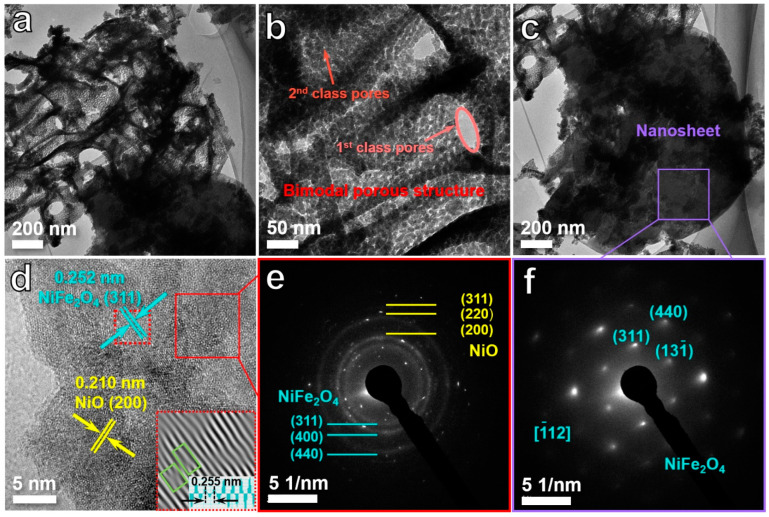
(**a**–**c**) TEM images of the D5 sample at different magnifications. (**d**) HRTEM image of porous ligament networks in the D5 sample, IFFT pattern and lattice spacing profiles of selected area (inset). The SAED patterns of porous ligament network (**e**) and nanosheet (**f**) in the D5 sample.

The microstructure of the D5 sample was analyzed in detail by TEM and high-resolution TEM (HRTEM). TEM images (Figure 2a–c) further demonstrate that the D5 sample was composed of nanosheet networks and ligament-pore networks. In addition, the ligament-pore network exhibits a typical feature of the bimodal porous structure with first-class pores of 50–150 nm and second-class pores of 3–5 nm (Figure 2a,b). The HRTEM image (Figure 2d) of the ligament-pore network shows the interplanar distance of 0.210 and 0.252 nm, which are consistent with the (2 0 0) planes of NiO and (3 1 1) planes of NiFe_2_O_4_, respectively [24,25]. In the meantime, as presented in the inset of Figure 2d, some obvious defects could be found at the lattice fringes by the inverse fast Fourier transform (IFFT). Specifically, in the (3 1 1) plane of NiFe_2_O_4_, the interplanar distance of the defect region was 0.255 nm, which is slightly higher than that of other normal regions and proves the existence of oxygen vacancies [26] in the lattice region of NiFe_2_O_4_. On the other hand, no obvious oxygen defects could be observed in the NiO region. Thus, the oxygen vacancies were proved to mainly come from NiFe_2_O_4_ rather than NiO in this study. The selected area electron diffraction (SAED) pattern of the ligament-pore network (Figure 2e) was obtained as concentric diffraction rings, which are attributed to (3 1 1), (4 0 0), and (4 4 0) planes of NiFe_2_O_4_ [27,28,29] and (2 0 0), (2 2 0) and (3 1 1) planes of NiO [24,30], suggesting a polycrystalline structure in the ligament-pore network. As shown in Figure 2f, the SAED pattern of the nanosheet is composed of some diffraction spots relating to (3 1 1), (1 3 −1), and (4 4 0) planes of NiFe_2_O_4_ with a matching zonal axis of [−1 1 2] [31]. This result indicates that the nanosheet is NiFe_2_O_4_ single crystal.

**Figure 3:**
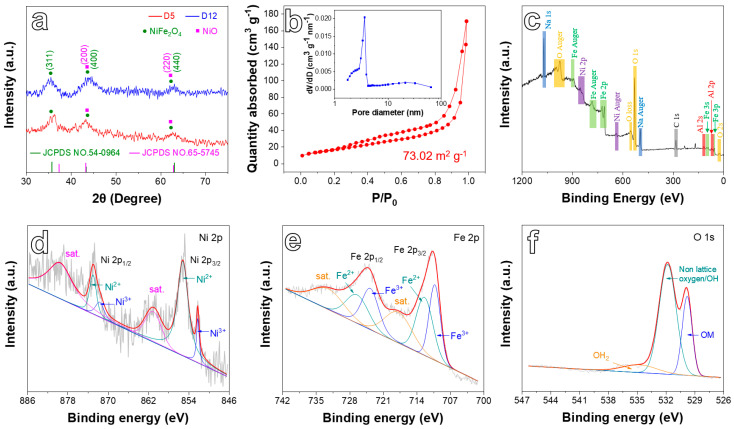
(**a**) XRD pattern of obtained samples. (**b**) N_2_ adsorption–desorption isotherm of the D5 sample and corresponding pore size distribution (inset). XPS spectra of the D5 sample: (**c**) survey spectrum, (**d**) Ni 2p, (**e**) Fe 2p, and (**f**) O 1s.

XRD tests were performed to analyze the phase composition of the products. As displayed in Figure 3a, the patterns included three strong diffraction peaks for each sample. According to JCPDS cards (NO. 54-0964, NiFe_2_O_4_; NO. 65-5745, NiO), the three diffraction peaks at 35.7°, 43.4°, and 63.0° relate to (3 1 1), (4 0 0), and (4 4 0) crystal planes of NiFe_2_O_4_ [29,32], and (2 0 0) and (2 2 0) crystal plane of NiO [33]. Moreover, the mass ratio of NiFe_2_O_4_ and NiO was close to 4.1:1 based on the quantitative calculation of XRD data by the software of JADE (MDI, Newtown Square, PA, USA). The above results demonstrate the co-existence of NiFe_2_O_4_ and NiO phases, corresponding to the TEM result well.

The N_2_ adsorption–desorption measurements (Figure 3b) indicate that the D5 sample exhibited a specific surface area of 73.02 m^2^ g^−1^, which was contributed to by the special porous network structure. While the specific surface area of D12 was 15.13 m^2^ g^−1^ (Figure S1), which is only 20.7% of that of D5, the curve in Figure 3b presents a type-III isotherm with an H3-type hysteresis loop. In addition, the inset shows that the pores of the sample ranged from 2 to 5 nm, demonstrating that the sample possesses abundant mesopores on porous skeletons that facilitated the transport and diffusion of electrons/ions and provided abundant electroactive sites for Li-ion storage [34,35].

Figure 3c–f discloses the surface details of the D5 sample by XPS. The survey spectrum (Figure 3c) confirms the existence of Fe, Ni, Al, O, and Na elements. The residual Al uncovered by weak peaks is a result of incomplete corrosion. In addition, the uncleaned caustic of NaOH remained on the specimen surface, resulting in a distinct XPS peak of Na. The Ni 2p spectra (Figure 3d) of the D5 sample included two main peaks (2p_3/2_ at 855.2 eV and 2p_1/2_ at 873.0 eV), along with two shakeup satellite peaks (861.2 and 879.8 eV). Although Ni^3+^ can be found at 852.2 and 871.7 eV, the relative atomic content of Ni^2+^ and Ni^3+^ was 83.6% and 16.4%, respectively. Thus, Ni^2+^ represents the main peak of the sample [25,35,36]. Fe 2p spectra (Figure 3e) possess two main peaks located at 712.0 (Fe 2p_3/2_) and 724.3 eV (Fe 2p_1/2_) [37,38]. The Fe 2p_3/2_ peak can be decomposed into two peaks at 712.6 (Fe^2+^) and 710.3 eV (Fe^3+^), while the Fe 2p_1/2_ peak can also be disassembled into two peaks at 726.8 (Fe^2+^) and 723.8 eV (Fe^3+^) [36]. The relative atomic content of Fe^2+^ and Fe^3+^ was 50.4% and 49.6%, respectively. This implies that the ratio of Fe^2+^ and Fe^3+^ on the surface of the material was close to 1:1. The O 1s spectra, as shown in Figure 3f, could be resolved into three peaks at 534.9, 531.8, and 529.8 eV, relating to the adsorbed water on the sample surface (H_2_O), adsorbed hydroxyl groups OH^−^, and non-lattice oxygen from the intrinsic oxygen vacancy on the surface [36], and O^2−^ in OM (M=Ni, Fe) bonds, respectively. Since the major sub-peaks include Ni^2+^ for Ni 2p, Fe^3+^ for Fe 2p, and non-lattice oxygen/OH for O 1s, the composition of the as-obtained D5 sample could be deduced to possess NiFe_2_O_4_ with oxygen vacancies. This result is consistent with the TEM analysis.

**Figure 4:**
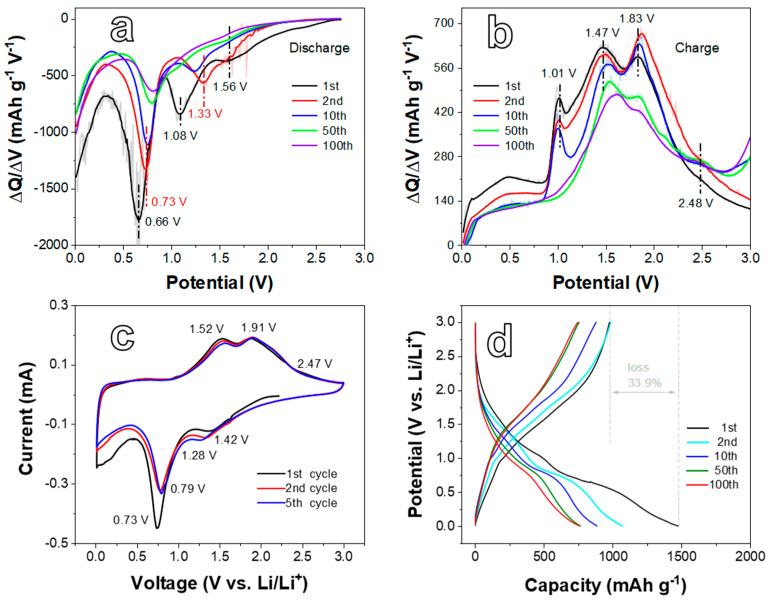
The differential capacity plots of the D5 electrode in the process of (**a**) Discharge and (**b**) Charge at the current density (CD) of 200 mA g^−1^. (**c**) CV curves of the D5 electrode at a scan rate of 0.1 mV s^−1^ in the voltage range from 0.01 to 3.0 V. (**d**) Galvanostatic charge/discharge profiles of the D5 electrode at the CD of 200 mA g^−1^.

**Figure 5:**
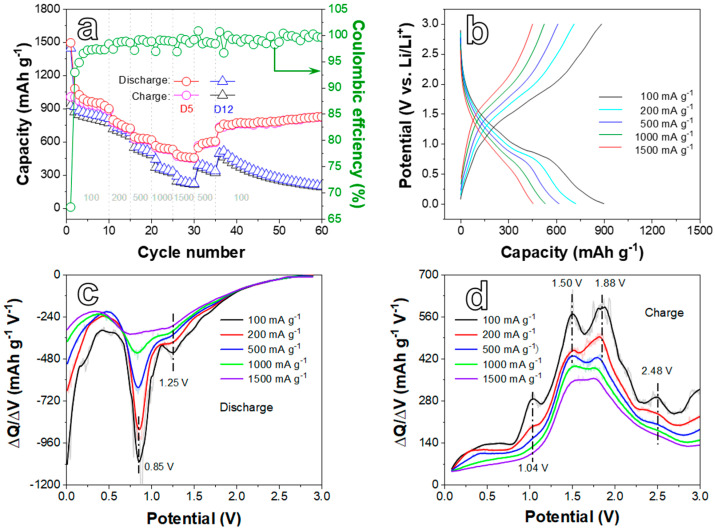
(**a**) Rate performances of D5 and D12 electrodes at different current densities. (**b**) Galvanostatic charge/discharge curves at 100, 200, 500, 1000, and 1500 mA g^−1^ of the D5 electrode, corresponding to the rate tests after five formation cycles at every current density. Differential capacity plots of the D5 electrode in the process of (**c**) Discharge and (**d**) Charge processes toward Figure 5b.

**Figure 6:**
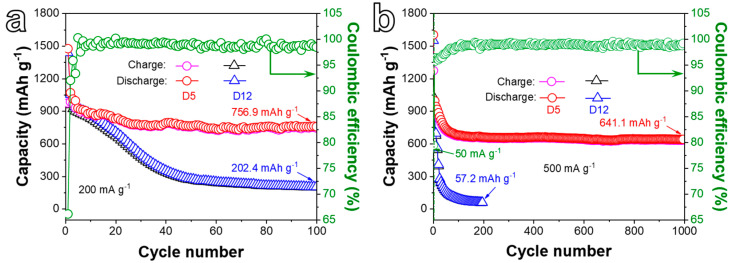
(**a**) Cycling performances of D5 and D12 electrodes at a CD of 200 mA g^−1^ for 100 cycles. (**b**) Cycling performances of D5 and D12 electrodes at 500 mA g^−1^ for 1000 cycles.

The electrochemical performances of the D5 and D12 anodes for LIBs are systematically investigated in Figure 4, Figure 5 and Figure 6. The differential capacity (DC) analysis and CV tests were applied to study the electrochemical reaction mechanism. DC plots in Figure 4a shows that during the first reduction process, the peak at 1.08 V represents the reduction in Ni^2+^ to metallic Ni, relating to the reaction NiO + 2e^−^ + 2Li^+^ → Ni + Li_2_O. While the peak located at 0.66 V relates to the reaction of NiFe_2_O_4_ from Fe^3+^ and Ni^2+^ to their metallic states, corresponding to NiFe_2_O_4_ + 8e^−^ + 8Li^+^ → Ni + 2Fe + 4Li_2_O [36]. It is worth noting that when NiFe_2_O_4_ was activated, both the Ni^2+^ and Fe^3+^ of NiFe_2_O_4_ were reduced at this stage, which is reflected in the CV and DC curves as a cathodic peak. In addition, the location of this cathodic peak was quite different from that of both NiO and Fe_2_O_3_ (more negative than that of NiO) due to the coordinated effect of Ni^2+^ and Fe^3+^ on NiFe_2_O_4_ [39]. Thus, NiO cannot be activated in this potential, and the reaction of NiO to Ni occurred with more positive potential. Furthermore, the appearance of the peak at 1.56 V can be ascribed to the insertion of lithium ions into NiFe_2_O_4_ [40,41]. In the subsequent cycles, the intensity of the reduction peaks at 0.66 and 1.08 V obviously decreased, and the peak potential moved slightly to the positive direction (0.73 and 1.33 V, respectively). This phenomenon can be attributed to the generation of a solid in electrolyte interface (SEI) layer and Li_2_O, as well as the irreversible reaction that occurred in the first reduction process. In the anodic process displayed in Figure 4b, the oxidation of Fe (1.47 V) and Ni (1.83 V) to Fe^3+^ and Ni^2+^, respectively, results in the formation of NiFe_2_O_4_ and the appearance of oxidation peaks. In addition, the weak peak at 2.48 V could be attributed to the oxidation of metallic Ni into Ni^2+^ to create NiO. These reactions are consistent with previous reports [41]. Furthermore, there also is a sharp peak at 1.01 V in Figure 4b, which may be the contribution of delithiation [42].

CV curves of the D5 electrode were measured at 0.1 mV s^−1^ in the voltage range of 0.01–3.0 V (Figure 4c). The CV curves reveal that reduction peaks at 0.73 and 1.42 V and oxidation ones at 1.52, 1.91, and 2.47 V can be found during the first cycle, which is consistent with DC plots. The peak around 1.08 V is not well shown due to the accuracy in CV curves. Moreover, the CV curves (Figure 4c) are well overlapped between the second cycle and the fifth cycle, revealing the good initial cyclic stability of the D5 electrode. Figure 4d presents the galvanostatic discharge/charge profiles of the D5 anode. Two voltage plateaus at around 1.0–1.8 and 0.4–0.8 V can be seen in the first discharge profile, matching the peak potential of CV curves. In the subsequent cycles, the plateaus shift to 1.2–1.5 and 0.6–0.9 V due to the creation of the SEI layer, complying with the analysis towards CV curves. The plateaus of the charge curves can be observed at 0.9–1.7 and 1.8–2.1 V when assigned to the corresponding oxidation peaks in CV curves. The initial discharge/charge capacity of the D5 electrode is 1475.6/975.5 mAh g^−1^ with a loss rate of 33.9%. The irreversible capacity loss is mainly related to the generation of the SEI film and Li_2_O and the decomposition of the electrolyte, which is common for TMO anode materials [35,43]. In addition, it can also be caused by the excess metallic Ni or NiO in the material. In the following cycles, the discharge capacities at the 2nd, 10th, 50th, and 100th cycles are 1070.3, 885.3, 764.2, and 757.0 mAh g^−1^, respectively. Though the discharge capacity of the subsequent cycles gradually declines, it still reaches 757.0 mAh g^−1^ after 100 cycles during cycling at a low current density.

The rate performance test of D5 and D12 electrodes was carried out at different current densities (CDs) ranging from 100 to 1500 mA g^−1^. The discharge/charge-specific capacities of the D5 electrode (Figure 5a) exhibit 899.3/884.4, 722.5/712.3, 617.5/610.4, 531.1/524.9, and 455.8/452.0 mAh g^−1^ at 100, 200, 500, 1000, and 1500 mA g^−1^, respectively. The electrode restores a stable discharge/charge capacity of 826.5/823.3 mAh g^−1^ after 60 cycles while the CD recovers to 100 mA g^−1^ again. Compared with the D5 electrode, the D12 electrode delivers lower discharge/charge-specific capacities at every CD. Moreover, the capacity decreases sharply and remains at 200.8/192.5 mAh g^−1^ when the CD backs to 100 mA g^−1^ after 60 cycles. Therefore, the D5 electrode exhibits a better rate of performance than the D12 electrode. The representative galvanostatic charge/discharge curves (Figure 5b) at different CDs further reveal the rate property of the D5 electrode. The shape of the profiles on different CDs is almost unchanged. As the CD increases, the curve shifts to the left. This moderate position change suggests that the material has an acceptable rate property. In Figure 5c,d, there are some peaks in DC plots at different CDs, which coincide with the analysis of peaks in Figure 4.

Figure 6a presents the cycling performances of D5 and D12 electrodes at 200 mA g^−1^ for 100 cycles. The first discharge/charge capacities of the D5 anode reach 1475.6/975.5 mAh g^−1^ with a Coulombic efficiency (CE) of 66.1%, and then a rapid discharge capacity fade can be observed at the beginning of the cycling test. This phenomenon is caused by the excess metallic Ni or NiO in the material and the reversible formation of the SEI layer due to electrolyte degradation [36]. In particular, the porous electrode with a high specific surface area can strengthen the electrolyte decomposition, leading to the formation of excessive SEI. Consequently, Li^+^ can be trapped and cause a large capacity loss at initial cycles [44]. Subsequently, and owing to the gradual activation of the active material, the specific capacity tends to be stable and remains at 756.9 mAh g^−1^ (0.91 mAh cm^−2^) after 100 cycles with a CE of 98.47%, suggesting good cycle stability. For the D12 electrode, however, the specific capacity fades rapidly, and the discharge specific capacity only retains 202.4 mAh g^−1^ after 100 cycles, which is obviously lower than the D5 electrode, implying the important role of ligament-pore networks in keeping the cycling stability of the electrode. Figure 6b displays the long-term cycling performance of D5 and D12 anodes at a higher CD of 500 mA g^−1^ for 1000 cycles. The electrodes were first activated at 50 mA g^−1^ for five cycles before cycling at 500 mA g^−1^ for the following cycles. The D5 electrode still retains a specific capacity of 641.1/634.6 mAh g^−1^ after 1000 cycles with a CE of about 99.0%. Nevertheless, the specific capacity of the D12 electrode decays sharply and only retains 57.2 mAh g^−1^ after 200 cycles, suggesting that the D5 anode possesses superior long-cycle performance than the D12 electrode at a relatively high CD.

**Figure 7:**
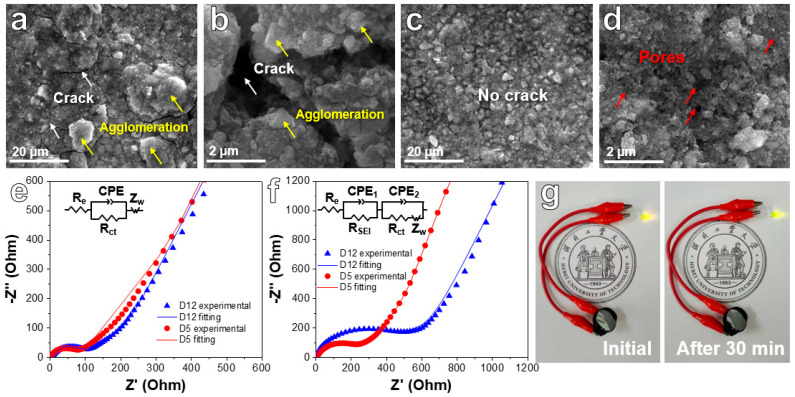
SEM images of the D12 electrode (**a**,**b**) and D5 electrode (**c**,**d**) after cycling for 100 cycles at a CD of 200 mA g^−1^. EIS tests and fitting curves of D5 and D12 electrodes in the fresh state (**e**) and after cycling for 100 cycles at 200 mA g^−1^ (**f**). (**g**) Digital photographs of an LED bulb powered by a battery with D5 anodes.

Figure S2 uncovers SEM images of fresh D5 and D12 electrodes. D12 presents some big particles (Figure S2a), while D5 reveals a plentiful porous structure (Figure S2b). Figure 7a–d reveals SEM images of the D5 and D12 electrodes after 100 discharge/charge cycles at 200 mA g^−1^. Some clear cracks can be observed in the D12 electrode after 100 cycles (Figure 7a,b). However, Figure 7c,d shows that there are no cracks in D5 electrode materials. In addition, the porous networks can still be found, revealing the remarkable structural integrity of the material during cycling. Although both D12 and D5 present a dual network structure, it is obvious that the nanosheet network in D12 occupies its dominant structure. However, the size of the nanosheet is too large compared with the bimodal porous network. After cycling, the nanosheets can easily form large agglomerations (Figure 7a,b, as shown by yellow arrows). This morphological feature makes it easy to cause the surface cracking of the electrode plate, resulting in the shedding of active materials from the surface electrode, which further leads to rapid and sustained capacity attenuation (Figure 6). In the D5 electrode, the bimodal porous network with a smaller nanosize occupies the dominant morphology. No obvious aggregates and cracks were found after cycling (Figure 7c,d). Its surface porous morphology can be well maintained. Therefore, its cycling performance remains stable (Figure 6). EIS tests were carried out and are shown in Figure 7e,f (inset: equivalent circuit model). EIS curves show a semicircle in high-frequency areas and are oblique in the low-frequency region, relating to charge transfer resistance and ion diffusion resistance, respectively. Though the EIS diagrams of two fresh electrodes show similar curve shapes and overlap with each other in certain content, the D5 electrode shows the lowest resistance after cycling, which can explain the good cycle stability of the battery in the long cycling process. Figure 7g reveals a light-emitting diode (LED) bulb powered by the D5 anode. After 30 min, the LED bulb still exhibited high brightness with a slight drop from its initial brightness, showing the great prospect of the dual-network NiFe_2_O_4_/NiO composites for the energy storage field.

Table 1 lists the Li storage properties of different NiFe_2_O_4_-based anode materials in LIBs [38,40,42,45,46,47,48]. It can be seen that the dual-network NiFe_2_O_4_/NiO composites present relatively good electrochemical performance in the listed studies. From the perspective of material composition, the as-prepared material in this paper is relatively simple and does not contain carbon materials such as graphene, carbon nanotubes, and so on. This helps increase the total capacity. From the perspective of material structure, the structural characteristics, including dual-network structure, high specific surface area, plentiful oxygen vacancies, and active sites, are conducive to the acquisition of high electrochemical performance. From the perspective of fabrication routes, the dealloying method is simple and facile. What makes sense is that no heat treatment is required during the preparation process, and thus the material in this paper exhibits better ecological and economic benefits. The good Li storage properties are related to the following points. Firstly, the dual-network structure provides sufficient buffer space for volume expansion during charge/discharge cycling, thus resulting in good cycling stability and good structural integrity. Secondly, second-class pores in bimodal porous structures can provide a facile transport pathway and a short distance for ion and electron movement, resulting in an improvement in cycling stability and rate performance. Thirdly, the highly specific surface area, rich porosity, and spinel structure with oxygen vacancies in the material provide highly conducting three-dimensional skeletons to improve conductivity and provide plentiful active sites to increase reaction kinetics. Therefore, the dual-network NiFe_2_O_4_/NiO composites exhibit excellent cyclic stability, rate performance, and a relatively high specific capacity.

## 3. Materials and Methods

### 3.1. Preparation of NiFe_2_O_4_/NiO Composites

**Figure 8:**
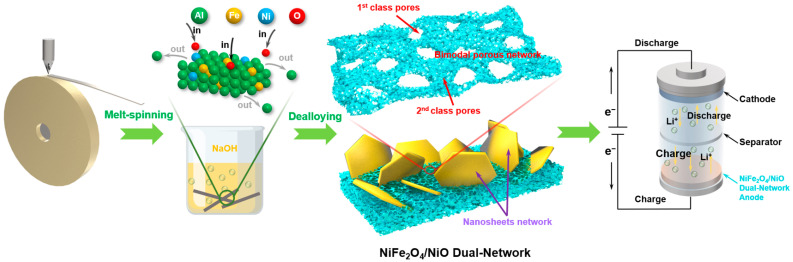
Illustration of the preparation process of NiFe_2_O_4_/NiO composites with a dual-network structure.

The typical preparation process of NiFe_2_O_4_/NiO composites is displayed in Figure 8. The Ni_1_Fe_3_Al_96_ ribbons were first prepared through the melt-spinning method (WK-II Melt Spinner, Physcience Opto-electronics Co., Ltd., Beijing, China) [19,22]. The nozzle diameter of the quartz tube was about 2 mm. The rotating speed of the copper roller was about 2200 r/min. The resulting ribbons were about 25 μm thick (Figure S3), 2.5 mm wide, and tens of centimeters long. Then, the dealloying treatment toward these ribbons (3 g for each experiment) was carried out in 500 mL 1.0 M NaOH solutions under an ambient atmosphere at 25 °C. According to the etching time, the dealloyed Ni_1_Fe_3_Al_96_ products were divided into two groups, D5 (5 h) and D12 (12 h), respectively. The dealloying time is determined according to experimental phenomena. After the dealloying reaction began, the sample reacted rapidly with the corrosive liquid to produce bubbles. As the reaction went on, the intensity of the bubble formation changed from intense to moderate. After 5 h of corrosion, the bubbles rarely spilled out, indicating that the main dealloying reaction was complete at this time. After 12 h of corrosion, there were no more bubbles overflowing at all, demonstrating that dealloying was fully complete. If the etching time was lower than 5 h, for example, 2 h (Figure S4), there was an obvious un-dealloyed area in the inner ribbon. Such a product contains a lot of tough solids with micron size, which cannot be used as anode material for LIBs. In this situation, 5 h and 12 h are the two important time nodes of the reaction in this experiment. During the corrosion, Al elements were preferentially leached out of the alloy due to the lowest potential of it in the three elements of the alloy, while the relatively noble Ni and Fe elements were self-assembled and oxidized into a NiFe_2_O_4_/NiO dual-network structure. The dual-network structure contains a nanosheet network and a bimodal porous network (Figure 8). The latter network possesses pores with two distinct scales, including first-class pores (50~200 nm) and second-class pores (5~10 nm). Finally, the materials were cleaned with ultrapure water and used for anode active materials.

### 3.2. Material Characterizations

The morphological and structural details of the as-obtained samples were examined using scanning electron microscopy (SEM, Nova nanoSEM 450, FEI, Hillsboro, OR, USA) and transmission electron microscopy (TEM, JEM-2010F, JEOL, Tokyo, Japan). The phase compositions of the samples were confirmed utilizing X-ray diffraction (XRD, D8 Advance, Bruker, Karlsruhe, Germany). The X-ray photoelectron spectroscopy (XPS, ESCALAB 250X, Thermo Fisher Scientific, Waltham, MA, USA) characterization of the D5 sample was performed to reveal information on superficial elemental compositions and states. In addition, a nitrogen adsorption–desorption isotherm was carried out (ASAP2020, Micromeritic, Norcross, GA, USA). The Brunauer–Emmett–Teller (BET) method was used to determine the specific surface area. The Barrett–Joyner–Halenda (BJH) method was also used to study the distribution of pore sizes.

### 3.3. Electrochemical Measurements

LIB anodes were obtained by mixing the as-fabricated products with Ketjen black (Aladdin, CAS number: 7440-44-0) and carboxymethyl cellulose (Aladdin, CAS number: 9004-32-4) (7:2:1, wt.%) in deionized water. The resulting slurry was overlaid onto a copper foil and then dried at 60 °C under a vacuum for 12 h. The mass loading of active materials was 1.0~1.2 mg cm^−2^. The electrode density was about 1.51 g/cm^3^ with an electrode porosity of about 51%. CR2032 coin-type cells were assembled in an inert glove box with argon protection using the as-prepared material and lithium foil. The electrolyte was 1.0 M LiPF_6_ (Aladdin, CAS number: 21324-40-3), which was dissolved in ethylene carbonate (EC, Aladdin, CAS number: 96-49-1) and dimethyl carbonate (DMC, CAS number: 616-38-6) (EC/DMC 1:1 by volume). The electrolyte loading in this study was about 250 μL for each coin cell. The electrochemical properties of electrodes were tested by a battery tester (CT-4008, NEWARE, Shenzhen, China) in a potential window from 0.01 to 3.00 V. Furthermore, cyclic voltammetry (CV) and electrochemical impedance spectroscopy (EIS) tests were carried out through an electrochemical workstation (IM6e, Zahner, Kronach, Germany).

## 4. Conclusions

In summary, a dual-network structural NiFe_2_O_4_/NiO anode was prepared through a facile dealloying technology. This special dual-network structure, consisting of a NiFe_2_O_4_ nanosheet network and NiFe_2_O_4_/NiO ligament-pore (bimodal porous) network, can be regulated in the volume ratio of two networks by adjusting the etching time. In particular, the bimodal porous network possessed pores with two distinct scales, including first-class pores (50~200 nm) and second-class pores (5~10 nm), providing a sufficient buffer space for volume expansion during the electrochemical cycling. The as-prepared material exhibited excellent electrochemical performance when used as a LIB anode, retaining a reversible capacity of 756.9 mAh g^−1^ at a current density of 200 mA g^−1^ after cycling for 100 cycles and 641.1 mAh g^−1^ at 500 mA g^−1^ after 1000 cycles. In addition, this material also presents an acceptable rate property. The good Li storage performance contributes to the special dual-network structure, which contains hierarchical porous features with pores in distinct scales and high specific surface area spinel structural NiFe_2_O_4_-based material with abundant oxygen vacancies and plentiful active sites. This work shows the potential application of dual-network materials in high-performance anodes for LIBs and may promote the development of spinel oxide anodes while also dealloying techniques in broad fields.

## Figures and Tables

**Table 1:** The comparison of electrochemical performances of NiFe_2_O_4_-based materials for lithium-ion batteries.

Anode Material	Electrode Mass Loading (mg cm^−2^)	Current Density (mA g^−1^)	Cycle Number	Reversible Capacity (mAh g^−1^)	Ref.
NiFe_2_O_4_/NC/N-doped graphene	1.5~2.0	100	50	963	[46]
NiFe_2_O_4_/graphene	–	100	100	732.79	[40]
NiFe_2_O_4_/carbon	1.0~1.5	200	200	676.6	[47]
NiFe_2_O_4_@C/CNTs	~2.0	200	100	1111.8	[45]
NiFe_2_O_4_/nickel cobalt double hydroxides	1.4	300	100	636.9	[42]
NiFe_2_O_4_/CNTs	–	500	100	406	[48]
NiFe_2_O_4_/NiO@Fe_2_O_3_ nanocubes	1.0~1.5	500	500	472.5	[38]
Dual-network NiFe_2_O_4_/NiO	1.0~1.2	200 500	100 1000	756.9 641.1	This work

## Data Availability

The data presented in this study are available on request from the corresponding author.

## Supplementary Materials

The following supporting information can be downloaded at: https://www.mdpi.com/article/10.3390/ijms24044152/s1.
