# Supplementary material for: Dealloyed Porous NiFe2O4/NiO with Dual-Network Structure as High-Performance Anodes for Lithium-Ion Batteries
Source: Int J Mol Sci. 2023 Feb 19;24(4):4152. doi: 10.3390/ijms24044152 (PMC9960563; doi:10.3390/ijms24044152)
Supplement: Supplementary file 1 [file ijms-24-04152-s001.zip › ijms-2150722-supplementary.pdf]

Supporting information

Dealloyed Porous $NiFe_2O_4$/NiO with Dual-Network Structure as High-Performance Anodes for Lithium-Ion Batteries

Chao Jin [1,2,†], Zigang Wang [1,†], Chang Luo [1], Chunling Qin [1], Yongyan Li [1,*] and Zhifeng Wang [1,2,*]

[1] School of Materials Science and Engineering, Hebei University of Technology, Tianjin 300401, China
[2] Key Laboratory for New Type of Functional Materials in Hebei Province, Hebei University of Technology, Tianjin 300401, China

Correspondence: liyongyan@hebut.edu.cn (Y.L.); wangzf@hebut.edu.cn (Z.W.)

† These authors contributed equally to this work.

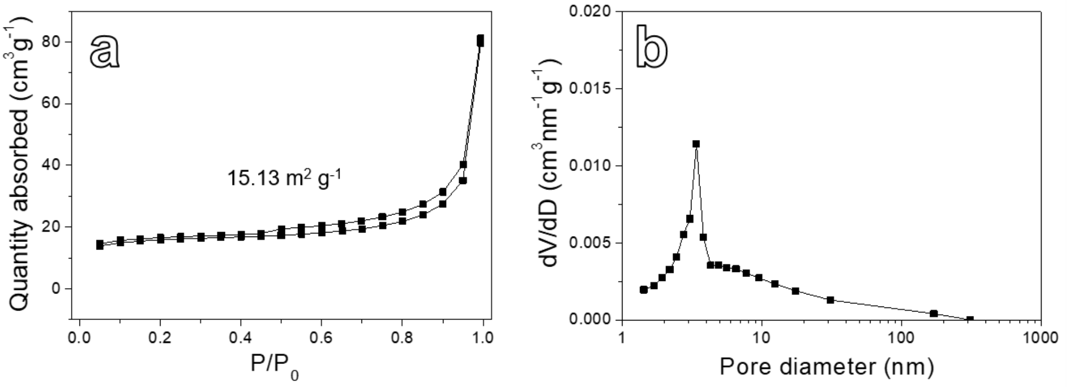

Figure S1. N_2 adsorption-desorption isotherm of the D12 sample (**a**) and corresponding pore size distribution (**b**)

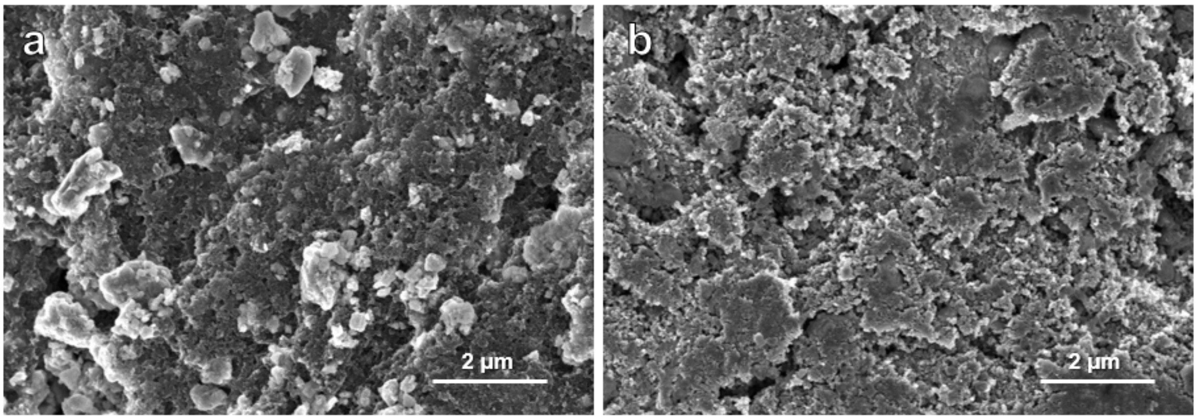

Figure S2. SEM images of the fresh electrodes：(a) D12. (b) D5.

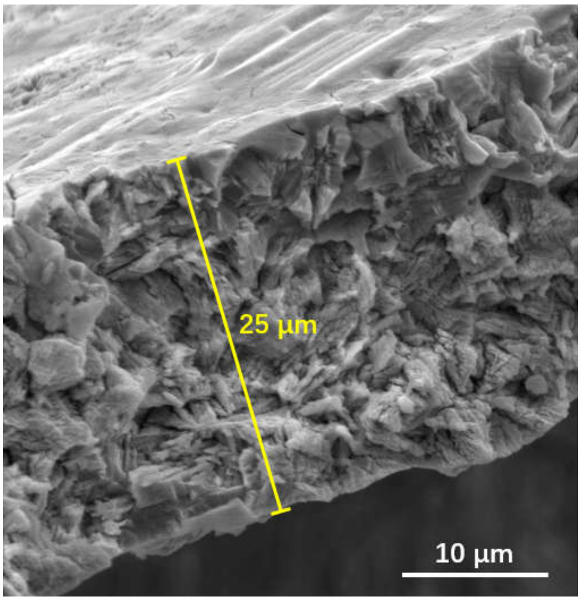

Figure S3. Cross-sectional SEM image of the original ribbon

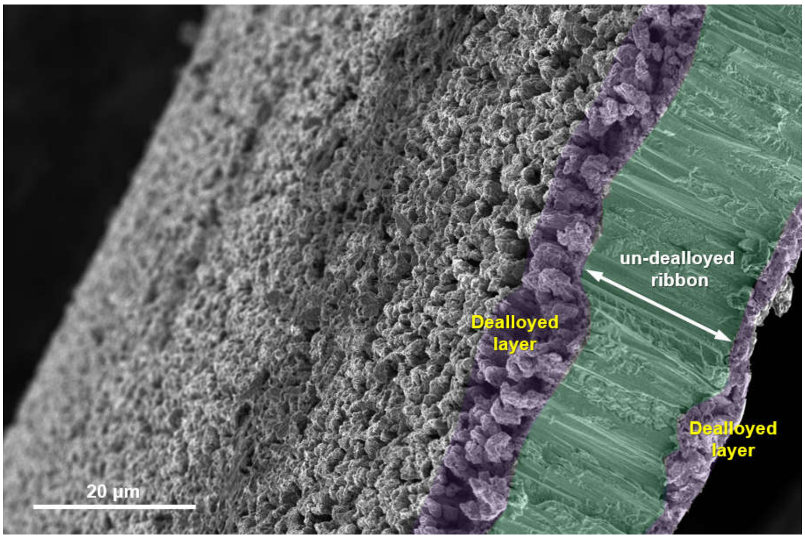

Figure S4. Cross-sectional SEM image of the dealloyed ribbon with etching time of 2 hours
